# FUS-mediated BBB opening leads to transient perfusion decrease and inflammation without acute or chronic brain lesion

**DOI:** 10.7150/thno.96721

**Published:** 2024-07-02

**Authors:** Sébastien Rigollet, Claire Rome, Thomas Ador, Erik Dumont, Chantal Pichon, Anthony Delalande, Emmanuel L. Barbier, Vasile Stupar

**Affiliations:** 1 Image Guided Therapy, Pessac, France.; 2 Univ. Grenoble Alpes, Inserm, U1216, Grenoble Institut Neurosciences, Grenoble, France.; 3 Université d'Orléans, LI²RSO, Orléans, France.; 4 ART ARNm, Inserm US55, Orléans, France.; 5 Laboratory of Experimental and Molecular Immunology and Neuromodulation (INEM), UMR 7355 CNRS-University of Orleans, Orleans, France.; 6 Institut Universitaire de France, Paris, France.; 7 Univ. Grenoble Alpes, Inserm, CHU Grenoble Alpes, CNRS, IRMaGe, Grenoble, France.

**Keywords:** focused ultrasound, MRI, neuroinflammation, secondary bioeffects, MR guidance

## Abstract

**Impact:** The permeabilization of the BBB to deliver therapeutics with MR-guided FUS redefines therapeutic strategies as it improves patient outcomes. To ensure the best translation towards clinical treatment, the evaluation of hemodynamic modifications in the CNS is necessary to refine treatment parameters.

**Methods:** MR-guided FUS was applied at 1.5 MHz with a 50 ms burst every 1 s to open the BBB. CBF, BVf and ADC parameters were monitored with MRI. Cavitation was monitored with a PCD during the FUS sequence and classified with the IUD index into three cavitation levels. We distinctly applied the FUS in the cortex or the striatum. After the BBB permeabilization, neuroinflammation markers were quantified longitudinally.

**Results:** The BBB was successfully opened in all animals in this study and only one animal was classified as “hard” and excluded from the rest of the study. 30 min after FUS-induced BBB opening in the cortex, we measured a 54% drop in CBF and a 13% drop in BVf compared to the contralateral side. After permeabilization of the striatum, a 38% drop in CBF and a 15% drop in BVf were measured. CBF values rapidly returned to baseline, and 90 min after BBB opening, no significant differences were observed. We quantified the subsequent neuroinflammation, noting a significant increase in astrocytic recruitment at 2 days and microglial activation at 1 day after FUS. After 7 days, no more inflammation was visible in the brain.

**Conclusion:** FUS-induced BBB opening transiently modifies hemodynamic parameters such as CBF and BVf, suggesting limited nutrients and oxygen supply to the CNS in the hour following the procedure.

## Introduction

To perform local drug delivery in the brain, the combination of focused ultrasound (FUS) with gas microbubbles (MBs) to permeabilize the blood-brain barrier (BBB) has gained interest since the pioneering demonstration by McDannold *et al*. in 2001 of the first extravasation of a gadolinium contrast agent (CA) in the central nervous system (CNS) 1. Prior to this, molecules such as hyperosmotic mannitol were used as agents to increase BBB permeability 2, albeit with non-specific effects on blood vessels, limited spatial control, and changes in permeability that were not robustly predictable. Conversely, the combination of FUS and MBs, guided by magnetic resonance imaging (MRI) and commonly referred to as MR-guided FUS (MRgFUS), offers a non-invasive and precisely controllable method to transiently permeabilize the BBB 2. Moreover, monitoring or even controlling cavitation can enhance the safety of the procedure 3-5. This method also enables the use of functionalized microbubbles for therapeutic applications. For example, microbubbles loaded with chemotherapy such as doxorubicin 6,7, paclitaxel 8, gemcitabine 9 or cationic microbubbles for nucleic acid delivery 10,11 have been developed, opening a new field of application.

However, the precise effects on brain tissue and vascularization after FUS-mediated BBB permeabilization with MBs have not been fully explored, which hinders its safe clinical translation. Early investigations by Hynynen *et al*. 12 in the late 1990s demonstrated that high-power FUS (6000 to 7000 W/cm²) without MBs substantially constricted the femoral artery, reducing blood flow by 50%. A return to the original state occurred within a week, although sometimes accompanied by localised haemorrhages. Gao *et al*. investigated the vascular effects of MBs coupled with 4.6 MPa high-intensity FUS on the liver with contrast-enhanced ultrasound. They concluded that it can temporarily block or reduce liver perfusion in rabbits, with a return to normal perfusion within one hour 13. Cho *et al*. monitored cerebrovascular dynamics in the cortex using intravital microscopy after BBB permeabilization with pressures ranging from 0.071 MPa to 0.25 MPa, revealing vasoconstriction or vasodilation in a small number of cases 14. In summary, these results suggest that FUS-mediated BBB permeabilization may affect cerebral blood flow (CBF).

Further reports corroborate this hypothesis. Following hind paw stimulation and BBB permeabilization using MBs and FUS in the right S1 cortex, arterial spin labelling (ASL) MRI measurements revealed a significant decrease in CBF changes within the right S1 cortex compared to the left 15. More recently, Stupar *et al.* 16 and Labriji *et al.* 17 reported a decrease in CBF following, respectively, FUS and non-focused US-mediated BBB permeabilization using MBs at about 0.38 MPa. It is therefore pertinent to inquire whether the reduction in CBF also occurs when BBB permeabilization is performed for drug delivery to the brain.

Several dysregulations of the CNS following FUS-mediated BBB opening have been reported. Kovacs *et al*. demonstrated that sterile inflammation was triggered in cases of BBB permeability at 0.3 MPa peak negative pressure (PNP) and noted an immediate damage-associated molecular pattern with IL-1, IL-18, and TNF-alpha transitory upregulation 18. Furthermore, angiogenic characteristics such as increased endothelial cell density and frequency of small blood vessel segments were observed several days after BBB opening. Additionally, blood vessel density exhibited a slight increase in the targeted area between 7 and 14 days following FUS treatment. These changes were followed by a return to baseline after three weeks 19. However, these cellular evaluations were not associated with an *in vivo* evaluation of brain perfusion.

We hypothesised that FUS-mediated BBB opening could lead to significant modifications in brain perfusion, thereby triggering cellular mechanisms to repair the BBB.

The primary objective of this study was to measure and compare changes in brain perfusion at various time points following FUS-mediated BBB permeabilization. Cavitation was monitored to assess the safety of the procedure and evaluate the deposited energy in the brain. MRI was used to quantify the CBF and the blood volume fraction (BVf) in the targeted brain regions at 30, 60, 90 min, and 24 h post-FUS. Histological changes in the brain tissue were examined with a focus on neuroinflammation markers, vessel integrity and signs of haemorrhages or oedema. This analysis was performed to detect any structural or cellular alterations resulting from BBB permeabilization.

## Methods

### Animal care and preparation

All animal procedures were conducted in compliance with ethical guidelines and were approved by both the Ethics Committee and the French Ministry of Research and Education (Authorization n° APAFIS#43777). Thirty-five male Wistar Han rats (age = 7 weeks) weighing between 270 and 350 grams were selected as experimental subjects and divided into three distinct groups. Anaesthesia was induced using 4% isoflurane in a mixture of oxygen and air (20/80%). Throughout the experimental protocol, anaesthesia was maintained at a consistent level between 1.5% and 2.5% isoflurane using the same oxygen and air mixture administered using an anaesthesia compact module (Minerve, Esternay, France). Respiratory rate and body temperature were maintained respectively around 60 breaths per min and 37 °C with care. Prior to the experiment, the hair on the top of the head was entirely removed using a trimmer followed by the application of a depilatory cream to optimise ultrasound transmission coupling. A 24 G catheter was carefully inserted into the tail vein to perform intravenous (IV) injections of saline, MBs, and MRI contrast agents (CA) (Figure 2A). At the end of the experimental procedures, the rats were closely observed until they fully regained consciousness, ensuring their well-being and recovery.

### Microbubbles

For this study, we used a solution of homemade lipidic-shelled MBs. The shell is composed of 1,2-distearoyl-sn-glycero-3-phosphocholine (DSPC) and 1,2-dimyristoyl-sn-glycero-3-phosphoethanolamine-N-[methoxy(polyethyleneglycol)-2000] (DSPE-PEG2000), 88:12 molar ratio. Lipids were obtained from Avanti Polar lipids. The gas core is composed of perfluorobutane (F2 Chemicals, UK). MBs have a mean diameter of 1.5 µm ranging between 1 and 5 µm, and concentration of 2×1010 MBs/mL (Figure 2B). MBs were activated in a 10 mM HEPES buffer (pH = 7.4) through 45 s of mechanical agitation using a VialMix® shaker (Lantheus Medical Imaging).

### MR-guided FUS

BBB permeabilization was performed using a dedicated MR-guided FUS system (Image Guided Therapy, Pessac, France) described by Magnin *et al*. 20. This setup includes a seven-concentric-elements transducer (Imasonic, Voray-sur-l'Ognon, France), which allows a control of the US focus length by steering (center frequency: 1.5 MHz, radius of curvature: 20.01 mm, focal length: 20 mm). The focal spot was characterized with a bullet hydrophone (HGL-0200, ±3 dB frequency range of 0.25-40 MHz; Onda Corporation, Sunnyvale, CA, USA) in a degassed separate water tank with a 3-axis LabVIEW controlled homemade system to position the hydrophone and measure the focal spot with a resolution of 0.1×0.1×0.2 mm^3^. The radial and axial lengths of the full width at half maximum (FWHM) were 1.1 mm and 6.6 mm, respectively (Figure S1A). The MR-guided FUS system includes an MRI saddle coil used for both transmission and reception. First, the position of the transducer within the MRI coordinate system was established. For this aim, an axial and a coronal, T1-weighted (T1-w), fast low angle shot (FLASH) images were acquired (TR/TE = 200/2 ms; 25 slices; field of view (FOV), 50×50 mm^2^; matrix, 128×128; voxel size = 391×391×1000 µm^3^; acquisition time (Tacq), 51 s). The location of the transducer was then computed offline using dedicated software from Image Guided Therapy (ThermoGuide®). An additional T2 TurboRARE sequence (TR/TE = 2500/33 ms; 15 coronal slices; FOV = 37×37 mm^2^; matrix, 256×256; voxel size, 145×145×1000 µm^3^; Tacq = 5 min) was used to precisely localize the target (Figure 2C).

Animals were placed under the ultrasound transducer with ultrasound gel to ensure proper coupling. A dose of 4×10^8^ MBs diluted in 100 µl HEPES buffer (10 mM, pH = 7.4) was injected over 10 seconds and flushed with 150 µL of saline. The ultrasound sequence started 15 s after the saline injection. Ultrasound stimulation consisted of 50 ms pulses with a pulse repetition frequency of 1 Hz (duty cycle = 5%) applied for 60 s. This duty cycle was not exceeded to avoid thermal elevation 21. We aimed to deliver an acoustic amplitude of 0.6 MPa PNP in the targeted brain region. Using the calibration of the ultrasound transducer (Figure S1B), the amplitude was adjusted for each animal depending on its weight and based on the attenuation of the brain (5 Np/m/MHz) and of the skull, according to Gerstenmayer *et al*. 22.

### Cavitation monitoring and analysis

Cavitation monitoring plays a crucial role in controlling the safety and in optimising the efficacy of BBB opening. In our study, we monitored cavitation with an unfocused passive cavitation detector (PCD) integrated at the center of the transducer (central frequency: 2 MHz, frequency bandwidth: 54% diameter: 20 mm; Imasonic, Voray-sur-l'Ognon, France). The PCD was connected to a digital oscilloscope (Picoscope 5242D, Pico Technology, UK) and signal acquisition was triggered by the ultrasound system (sampling frequency, 15.625 MHz). Data were analysed offline to assess the safety of the BBB opening with a Python script (version 3.10.12, Python Software Foundation, USA). Recently, Novell *et al*. 4 proposed a new cavitation monitoring method that does not require the acquisition of a baseline signal prior to the MBs injection, allowing the characterization of inertial cavitation events using a shorter time range, leading to a better estimation of the acoustic energy deposition. Stable cavitation is characterised by oscillating microbubbles that do not collapse, typically driven by a low amplitude acoustic field. In contrast, inertial cavitation involves the collapse of MBs, producing broadband acoustic emissions. In this context, the appearance of ultra-harmonic frequencies serves as a common marker for the onset of inertial cavitation 23. The monitoring of this phenomenon involves the calculation of the intrapulse ultra-harmonic dose (IUD) and intrapulse harmonic dose (IHD) 4. These were calculated considering respectively 1.5×f0, 2.5×f0, 3.5×f0 and 2×f0, 3×f0, 4×f0 frequencies with a 50 kHz bandwidth. Each 50 ms pulse was analysed as follows: an initial pulse cut of 200 µs was set to get rid of non-linear oscillations of the transducer, followed by 247 windows of 200 µs taken into account for further calculations of IUD and IHD, and the final 400 µs were not analysed to also avoid distortions. A separate fast Fourier transform was performed on the 247 windows before calculating IUD and IHD. It is important to note that a risk of inertial cavitation events can occur if the IUD index increases rapidly. The cavitation signal was computed as follows:



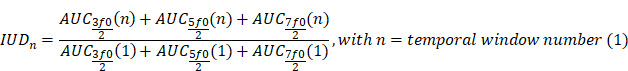



We defined a threshold likely to represent a strong destabilization of the microbubbles and therefore induce harmful effects on the brain tissue. We set this threshold to 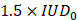. When the value of 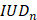 exceeded 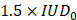, we classified the cavitation signal as inertial. We then evaluated the number of consecutive events exceeding 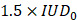 (the black dashed line in Figure 2E). Previous studies have evaluated different levels of BBB opening as the acoustic energy strongly influences the outcome of BBB opening 5,24,25. We classified the animals in our study into three different levels of cavitation dose: soft, mild, and hard. The soft case was considered when no event exceeded the settled cavitation threshold (Figure 5A.5), the mild case when most events were between the noise threshold and some events exceeded the threshold (Figure 5B.5), and the hard case when events exceeded the threshold and many of them were consecutive (Figure 5C.5) and associated with many red blood cell extravasation (Figure 5C.3).

### Study design

**Figure 1:**
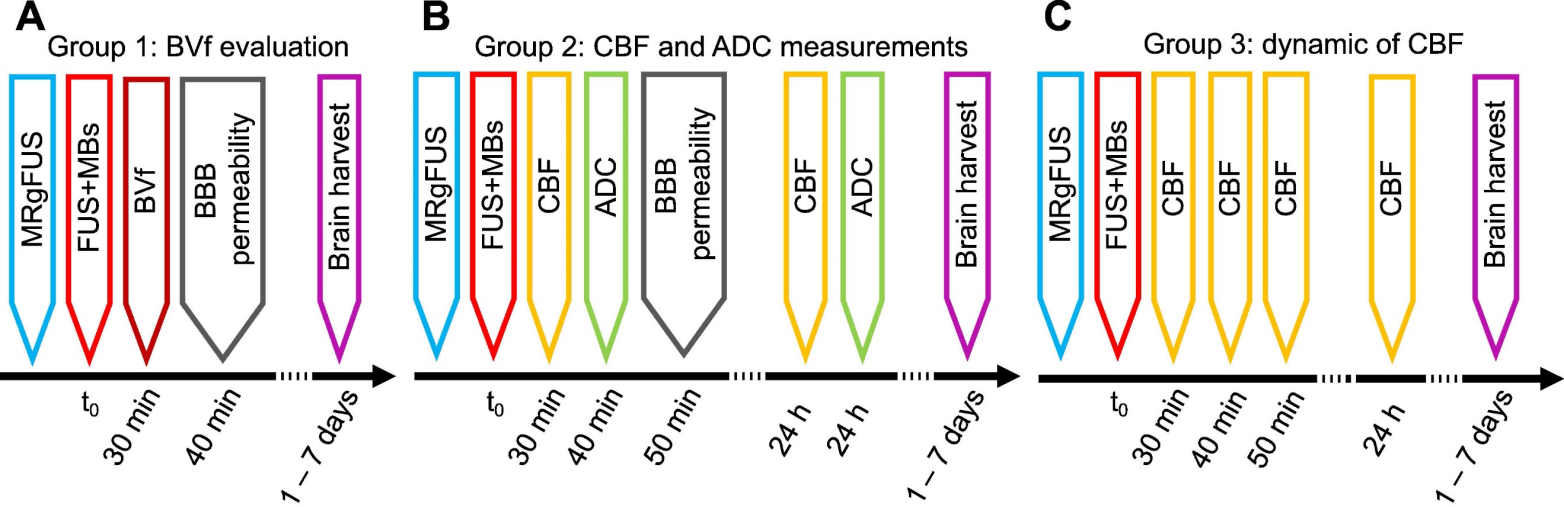
Workflow of the study, with the timeline of each group.

This section describes the groups of animals and the MRI measurements performed after MRgFUS BBB opening (Figure 1). A detailed description of each MRI sequence is provided in section 2.6.1. Histological analyses were performed at the end of the procedure (see 2.7). As the evaluation of CBF and ADC requires that subjects are free of contrast agents, measurements of BVf, which require USPIO contrast agents, cannot be performed at the same time points.

#### Group 1: CBF and ADC evaluation

Group 1 (n = 14) was used to evaluate the effects of BBB opening localization on CBF and ADC 30 min after FUS. A second imaging session was performed 24 h after FUS to measure these parameters prior to analysis. Half of the animals underwent FUS in the cortex, while the other half underwent FUS in the striatum. Electronic steering of the transducer was used to modify the position of the focal point, thereby adjusting the target location (see 2.3). This allowed assessment of whether changes in CBF or ADC were dependent on the target location.

#### Group 2: CBF over time

Group 2 (n = 14) was designed to measure the evolution of CBF at 30, 60, 90 min, and 24 h after BBB opening. To avoid any bias in our CBF data, no Gd-DOTA injection was performed in group 2 and therefore BBB opening could not be evaluated.

#### Group 3: BVf evaluation

Group 3 (n = 9) was used to study the BVf 30 min after BBB opening. An anatomical T2-weighted (T2-w) image and multi-gradient echo images were acquired before and after injection of USPIO nanoparticles (200 µmol iron/kg, Synomag-D 50 nm, Micromod, Rostock, Germany) to measure BVf. To assess BBB opening, a T1-w image was acquired before and after injection of Gd-DOTA (200 µmol/kg, DOTAREM®, Guerbet, Villepinte, France). A second imaging session was performed one week after FUS to assess the presence of brain haemorrhage using multi-gradient echo images and the integrity of the BBB using T1-w images and an injection of Gd-DOTA. In this group, 6 animals received FUS treatment in the cortex and the last 3 in the striatum.

### Magnetic resonance imaging (MRI) data and analysis

#### CBF, ADC, BVf and BBB opening area measurements

**Figure 2:**
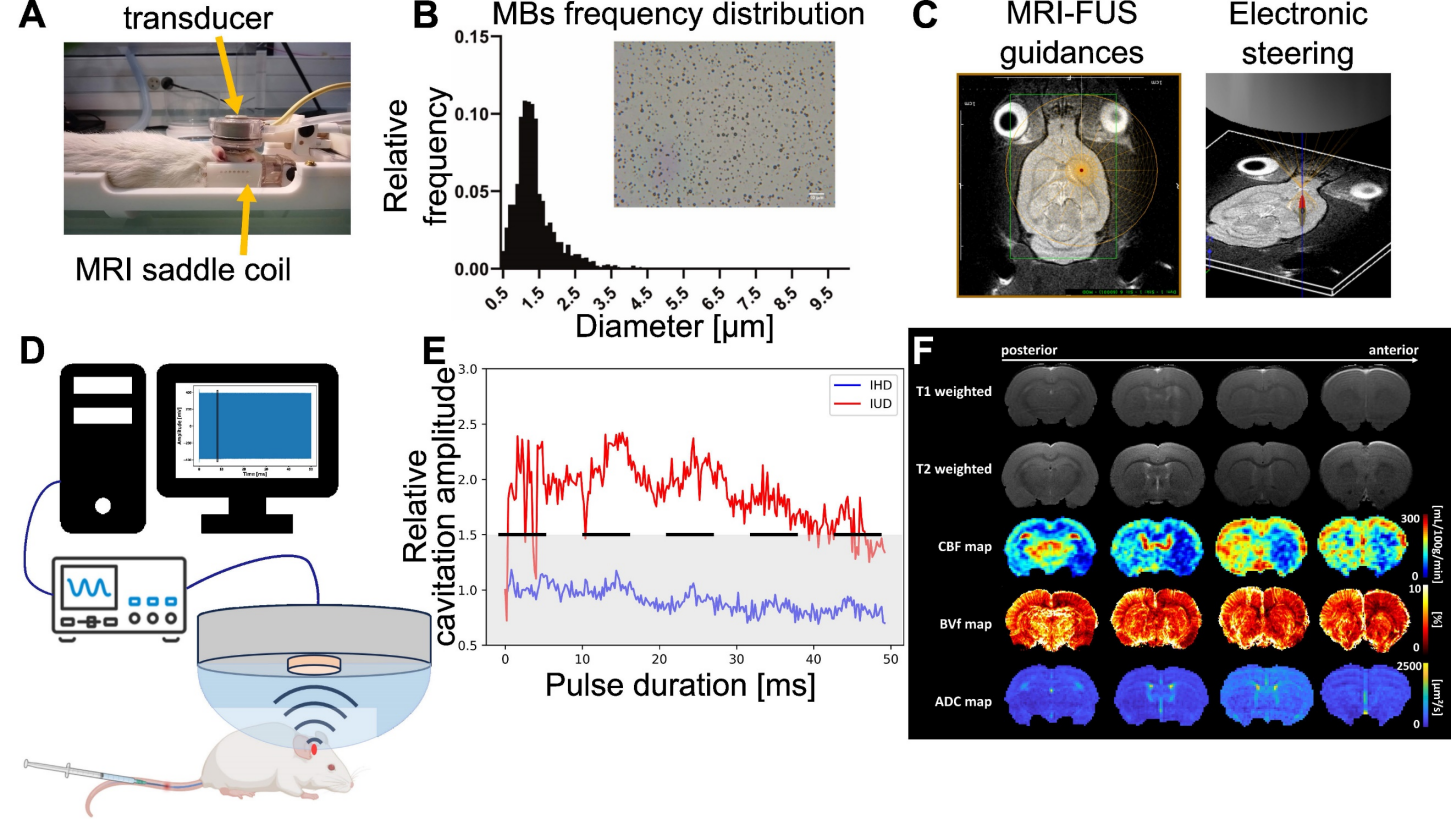
Outline of the different steps of the study. The animal is placed in the MRgFUS bed, which includes an MRI saddle coil with the transducer placed over the animal's head (**A**). the region of interest is targeted using Thermoguide® software and focal steering (**B**). Micrograph showing MBs and histogram of microbubbles diameter distributions (**C**). Cavitation monitoring setup with the PCD in the center of the transducer, an oscilloscope and signals processed by a computer (**D**) to calculate IHD and IUD for each pulse duration. An example of the IHD and IUD evolution over a single pulse duration is shown, with the IUD evolution in red, the IHD evolution in blue, the black dashed line representing the inertial threshold, and the grey dashed area indicating the noise level (**E**). Representative MRI images and parametric maps obtained from one animal in this study (**F**).

MRI was performed at 4.7T (Avance III, Bruker, Germany) at IRMaGe MRI facility (Grenoble, France). The scanner was equipped with a volume transmit coil and a single-element surface receive coil. An overview of the different MRI images acquired in this study is presented in Figure 2F. Anatomical T2-w images were acquired using a TurboRARE sequence (TR/TE = 2500/33 ms; 23 axial slices; FOV = 30×30 mm^2^; matrix, 256×256; voxel size, 117×117×1000 µm^3^; RARE-factor = 8; Tacq = 2 min 40 s). CBF was quantified as described by Hirschler *et al*. 26 and using:

• An inversion efficiency map obtained from a 1 mm-thick slice located 5 mm downstream of the pseudo-continuous arterial spin labelling (pCASL) plane and acquired with a flow-compensated gradient-echo sequence (TR/TE = 225/3.6 ms; FOV = 30×30 mm^2^; matrix, 256×256; voxel size = 117×117×1000 µm^3^; Tacq = 3 min 30 s);

• A T1 map of the brain tissue derived from a nonselective inversion recovery sequence (TR/TE = 10000/19 ms; 18 inversion times (TIs) between 30 and 10000 ms; FOV = 30×30 mm^2^; matrix, 128×128; voxel size, 234×234×1000 µm^3^; Tacq = 4 min);

• A perfusion-weighted signal obtained with a pCASL-encoded echo-planar imaging (EPI) sequence (TR/TE = 4000/21 ms; eight axial slices; FOV = 30×30 mm^2^; matrix, 128×128; voxel size, 234×234×1500 µm^3^; number of repetitions, 30; label duration, 3000 ms; post labelling delay, 300 ms; Tacq = 4 min).

To detect oedema in the brain, diffusion tensor imaging (DTI) EPI scans were acquired and ADC maps were computed (TR/TE = 2300/21 ms; b-values = 0 and 1000 s.mm^-2^; 30 directions; 23 axial slices; FOV = 30×30 mm^2^; matrix, 128×128; voxel size, 234×234×1000 µm^3^; Tacq = 10 min 44 s). BVf was derived from multi gradient echo (MGE) images acquired before and after the injection of USPIO (TR = 920 ms; TE1 = 3.5 ms, ΔTE = 5 ms; number of echoes, 8; 19 axial slices; FOV = 30×30 mm^2^; matrix, 256×256; voxel size, 137×137×1000 µm^3^; Tacq = 5 min 53 s).

#### MRI processing

ADC maps were reconstructed using ParaVision 6.0.1 (Bruker, Ettlingen, Germany). All MRI data were processed and analysed using MP3 27. Raw T2-w images of the brains were masked using pulse-coupled neural networks 28 and registered to a rat brain template 29 using *flirt* from FSL software 30. The resulting transformation was then applied to each scan acquired during the session. BVf maps were calculated from the change in transverse relaxation rate due to USPIO as described by Troprès *et al*. 31. Data from the ipsilateral (i.e., the hemisphere that underwent FUS) and contralateral cortex and striatum were extracted using the SIGMA rat brain atlas 32 (https://www.nitrc.org/projects/sigma_template). We calculated the difference between T1-w images obtained before and after Gd-DOTA injection and manually delineated the BBB-open area.

### Histological analysis

#### Immunohistochemistry staining protocols

Brains were quickly removed, frozen in -40°C isopentane and stored at -80°C. Coronal slices (10 µm) at various points along the anteroposterior axis were obtained with a cryotome operating at -20°C. Haematoxylin and eosin (H&E) staining and immunohistochemistry for vascular and neuroinflammation markers were performed.

For immunohistochemistry, sections were fixed in paraformaldehyde (Fluka Chemie AG, Buchs, Switzerland). Brain sections were then incubated overnight at 4°C in rabbit anti-GFAP (1:1000, DAKO, 70334), rabbit anti-Iba1 (1:500, Abcam, ab108539) or mouse anti-SMI 71 (1:1000, Biolegend Cat., No. 836803). Sections were then incubated with goat anti-rabbit (1:1000, Invitrogen, A11008) or donkey anti-mouse (1:1000, Thermofisher, A31540) secondary antibody. After rinsing in PBS-Tween 0.1%, coverslips were applied on slides with the nuclear marker DAPI (Thermofisher).

#### Image acquisition and analysis

Images were digitised using the Axioscan scanner (Zeiss, Oberkochen, Germany) and analysed using ZEN 3.6 software (Zeiss, Oberkochen, Germany), QuPath 33 and Python 3.10.0. On H&E images, the entire section was visually inspected, with a special attention to the application of FUS, to look for changes. Red blood cells (RBCs) extravasations were analysed by counting the number of sites with at least 5 RBCs. For immunofluorescence images, regions of interest (ROI) were manually drawn to extract the treated and control regions using ZEN software. Iba1 quantification was performed using QuPath with microglial cell segmentation by pixel classification and mean fluorescence intensity extracted for quantification. Astrocytes were quantified using a Python script by retrieving the fluorescent fraction of the surface area and mean fluorescence intensity. Similarly, vessel quantification was performed using a Python script that retrieved the mean vessel diameter, length and tortuosity in the contralateral, ipsilateral and two adjacent areas of the ipsilateral region.

### Statistical analysis

Statistical analysis was performed using GraphPad Prism 9.0 software (GraphPad Software Inc., La Jolla, CA, USA). Blood volume, flow and water diffusion data are presented as mean ± standard deviation (S.D.). Histological quantifications are expressed as a percentage of area (%) and mean fluorescence intensity (arbitrary unit (a.u.)). A non-parametric two-tailed paired t-test was used for pairwise comparisons (α level was set at 0.05).

## Results

### MRgFUS BBB opening effectiveness

**Figure 3:**
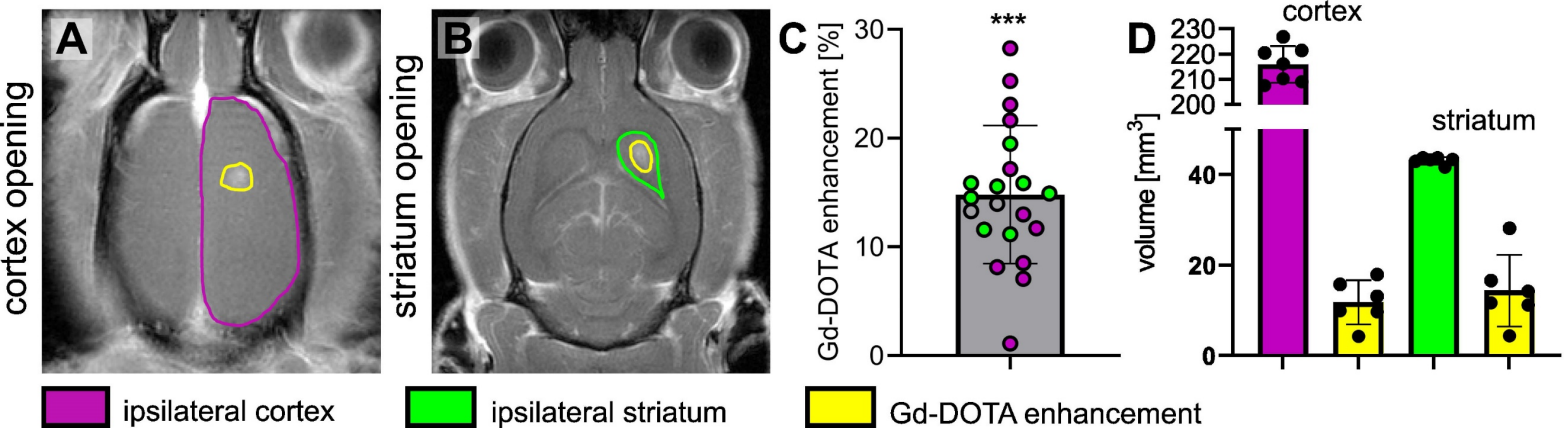
Representative T1-w images of the brain after the BBB has been opened at the level of the cortex (outlined in purple) (**A**) and the striatum (outlined in green) (**B**) and Gd-DOTA injection, the opened BBB is outlined in yellow. (**C**) Relative Gd-DOTA enhancement of the ipsilateral region after Gd-DOTA injection. In purple, the subjects targeted in the cortex and in green the ones targeted in the striatum. A two-tailed non-parametric paired t-test (***p < 0.001) was performed on the ipsilateral region between images acquired before and after Gd-DOTA injection. (**D**) The volume of the ipsilateral cortex (purple), ipsilateral striatum (green) and the corresponding volume of the BBB opening for subjects targeted in the cortex and striatum. The results are expressed as mean ± S.D.

The MRgFUS system was integrated with the MRI to precisely guide the focal point to the region of interest. We targeted two different brain regions: the cortex (located 0.5 mm posterior and 3 mm lateral to the bregma on the right side of the skull and at a depth of 1.5 mm from the brain surface) and the striatum (0.5 mm posterior and 3 mm lateral to the bregma on the right side of the skull and at a depth of 5 mm from the brain surface) (Figures 3A-B). The BBB was successfully opened in all animals, with an average enhancement of 18.66% following IV injection of Gd-DOTA (Figure 3C). We compared the volume of Gd-DOTA extravasation with the volume of the targeted brain regions (Figure 3D). Altogether, 5% of the cortex volume and 30% of the striatum were permeabilized.

### Safety of the procedure: passive cavitation monitoring, MRI images, and HE analysis

**Figure 4:**
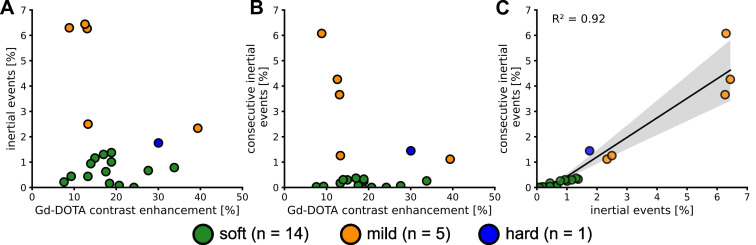
Number of events above the 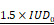 threshold (i.e. inertial events) (**A**). Number of consecutive inertial events (**B**). Evolution of the consecutive inertial events depending on the total number of inertial events and linear regression (**C**). The values are expressed as a percentage of the total number of IUD_n_ measured.

Cavitation was closely monitored during the application of the FUS sequence. IUD and IHD indices were calculated to provide insight into the mechanical aspects of BBB opening. Subjects were categorized into three levels of cavitation intensity - soft, mild, and hard - based on the IUD level and the inertial threshold equal to 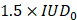. The IHD parameter did not vary enough to discriminate between the subjects in this study. This categorization process allowed us to exclude animals that received excessively intense energy deposition. Twenty-three animals from groups 1 and 3 were monitored with the PCD and only twenty were analysed due to technical issues. We quantified both the number of cavitation events that exceeded the inertial threshold and the number of consecutive inertial events. We saw an increase in the number of inertial events and the number of consecutive inertial events that did not correlate with the contrast enhancement measured in MRI (Figure 4A and Figure 4B) suggesting that MRI-based quantification has limitations. A linear fit between the consecutive and total inertial events showed a good correlation (R² = 0.91) and seems to discriminate two main groups. Fourteen animals (70%) were classified as soft and 6 as mild or hard (30%). We performed H&E analysis to validate the correlations.

**Figure 5:**
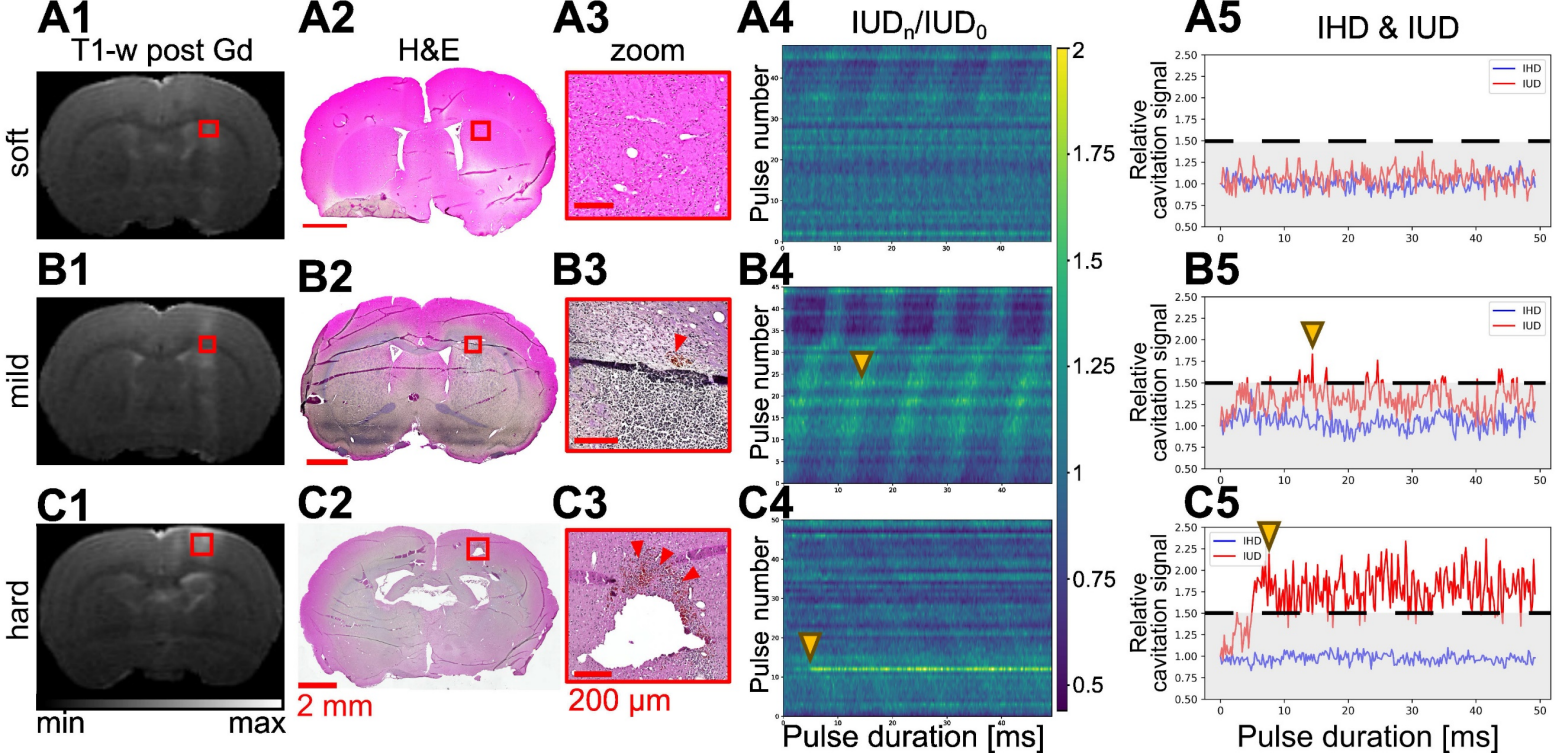
Representative examples of soft (row **A**), mild (row **B**), and hard (row **C**) BBB opening with the MRI T2-w image (**A1-C1**), micrograph of H&E staining (**A2-C2**), and a zoom on the treated area (**A3-C3**). Associated evolution of relative IUD over the 50 ms ultrasound bursts for each pulse of the sequence (**A4-C4**) and the evolution of IUD and IHD for a single pulse (**A5-C5**) extracted from column 4.

In the soft cases, the relative cavitation signal values did not show any significant humps (Figure 5A.4), and the IUD remained within the noise level, as shown in Figure 5A.5. No erythrocytes were visible on H&E images (Figures 5A.2-3). Inertial and consecutive inertial events did not exceed 1.4% and 0.3%, respectively, as shown in Figure 4A and Figure 4B.

When analysing the other subjects, we observed more events exceeding the inertial threshold (Figures 5B.4-5). This increase was associated with an increase in the number of total and consecutive inertial events, reaching respectively almost 6.3% and 6%. In the permeabilized BBB region (Figure 5B.1), a few erythrocyte extravasations were visible on H&E (Figures 5B.2-3). We noted that for the soft and mild cases the average cavitation signal did not exceed the inertial threshold (Figure S2).

Only one animal was excluded from the mild group because of too many erythrocytes present on the H&E staining (Figures 5C.2-3). We classified this subject as hard and it was excluded from the rest of the study. It was characterised by a sudden bump at the beginning of the FUS sequence (Figures 5C.4-5) that exceeded the inertial threshold (beginning highlighted with yellow arrow in Figures 5C.4-5) and remained above the threshold until the end of the pulse. On average, for the whole duration of the pulse, the cavitation signal remained above the threshold (Figure S2.C2). Note that this behaviour was measured in one subject only, as our study did not aim to evaluate critical cavitation situation. This observation implies the beginning of a haemorrhage and therefore a change in the concentration of the microbubbles, as evidenced by the decrease in cavitation signal for the following pulses. The T1-w image (Figure 5.C1) showed a large extravasation of Gd-DOTA as well as several RBCs (Figures 5C.2-3). We counted more than 100 RBCs sites (Figure S3).

### Characterization of hypoperfusion after BBB opening in the striatum and in the cortex

To determine the effect of FUS combined with MBs on CBF, pCASL datasets were acquired 30 min after delivering the treatment. FUS targeted the striatum in one group and the cortex in the other, with precise targeting achieved through MRI guidance.

**Figure 6:**
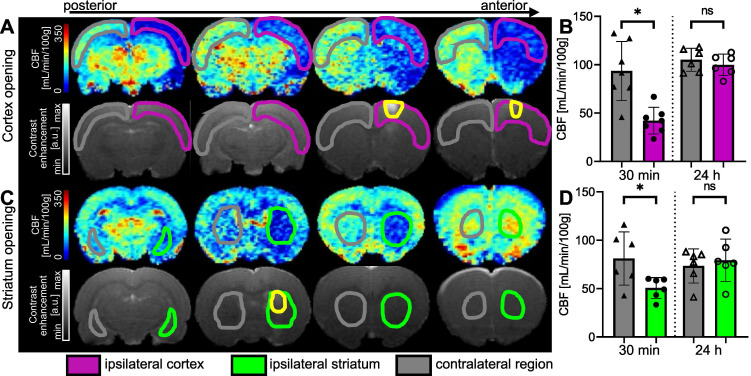
Examples of CBF maps 30 min after BBB opening and corresponding T1-w images after Gd-DOTA injection for cortex (**A**) and striatum (**C**) permeabilization. The area with opened BBB is outlined in yellow. CBF 30 min and 24 hours after FUS in cortex (**B**) and striatum (**D**). The data were analysed using a two-tailed non-parametric test (Wilcoxon) (*p < 0.05; **p < 0.01; ***p < 0.001; ns, non-significant). The results are expressed as mean ± S.D.

When the right cortex was targeted (Figure 6A), we observed a large decrease in perfusion in the treated cortex. FUS-induced BBB opening led to a decrease in perfusion that extended beyond the treated area. After 30 min, the CBF in the treated cortex decreased by 54%. An average CBF of 42.24 ± 13.8 mL/min/100g (n = 7) was measured in the ipsilateral cortex compared to 93.57 ± 30.5 mL/min/100g in the contralateral one (Figure 6B), showing a significant difference between both regions (*p* = 0.0156). After 24 hours, there was no significant difference in CBF between the ipsilateral and contralateral cortices.

When targeting the striatum (Figure 6C), we observed a decrease in CBF values at the targeted location and even beyond, according to the anterior CBF maps shown. The quantifications in Figure 6D show a 38% reduction in perfusion in the targeted striatum compared to the contralateral striatum 30 min after BBB opening. The difference between the two striatum is significant (*p* = 0.0313), with a mean CBF of 50.68 ± 10.8 mL/min/100g in the ipsilateral striatum and 81.21 ± 27.5 mL/min/100g in the contralateral striatum. In this case, the decrease in perfusion seems to be limited to the single target region, as no decrease in perfusion was observed in the cortex. After 24 h, the difference between the two striatum is no longer significant.

**Figure 7:**
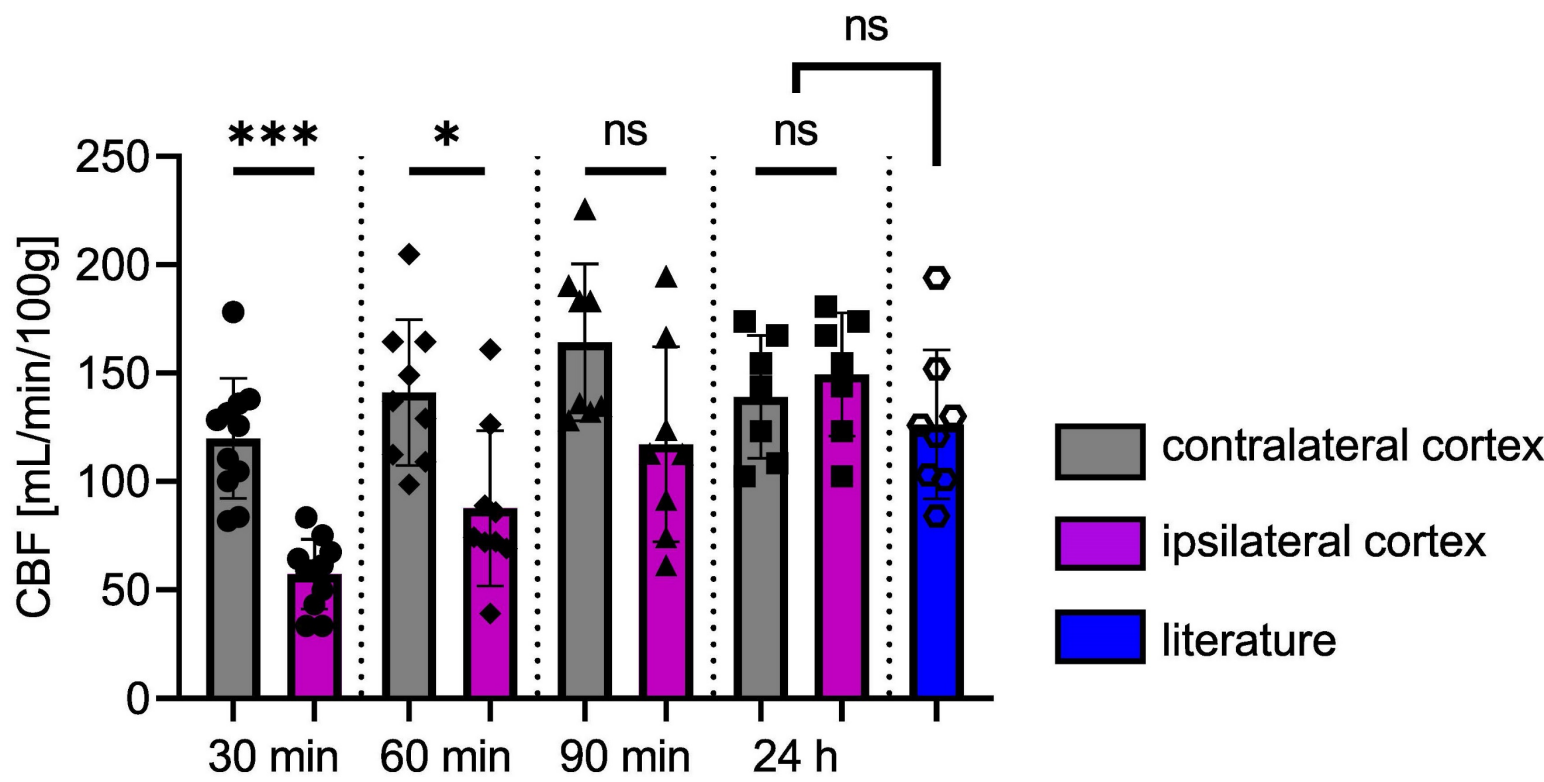
CBF in ipsi- and contralateral cortices as a function of time following the opening of the BBB. Values from the literature, obtained under experimental conditions similar to ours but without FUS, represent the normal CBF values (blue). The data were analysed using a two-tailed non-parametric test (*p < 0.05; **p < 0.01; ***p < 0.001; ns, non-significant). The results are expressed as mean ± S.D.

Figure 7 shows the time course of CBF. There is a significant difference between the two cortices at 30 min (*p* = 0.0010) and 60 min (*p* = 0.0273) after FUS. At 90 min post-FUS, the difference is no longer significant (*p* = 0.0547, CBF_ipsi_ = 117.2 ± 44.9 and CBF_contra_ = 164.2 ± 36.2 mL/min/100g). After 24 h, CBF returns to baseline values, showing no significant differences between the two cortices, consistent with observations 24 h post-FUS in group 1. For comparison, values from the literature (34-39) obtained in the brains of rats anaesthetised with isoflurane (n = 8) are shown in Figure 7. There is no significant difference between our data and those from the literature at 24 h. The reduction in CBF in the targeted cortex is transient, as it takes 90 min after BBB opening for differences between the two cortices to become non-significant following the initial significant decrease.

ADC maps acquired 30 min and 24 h after BBB opening could provide insight into a possible oedema resulting from the FUS procedure (Figure S4). No significant difference was measured in either the cortex or the striatum at either time point. The slight increase measured at 24 h is related to the repeatability measurement, as the increase is global and not attributable to the spread of oedema throughout the brain.

### Assessment of BVf 30 minutes after BBB disruption

**Figure 8:**
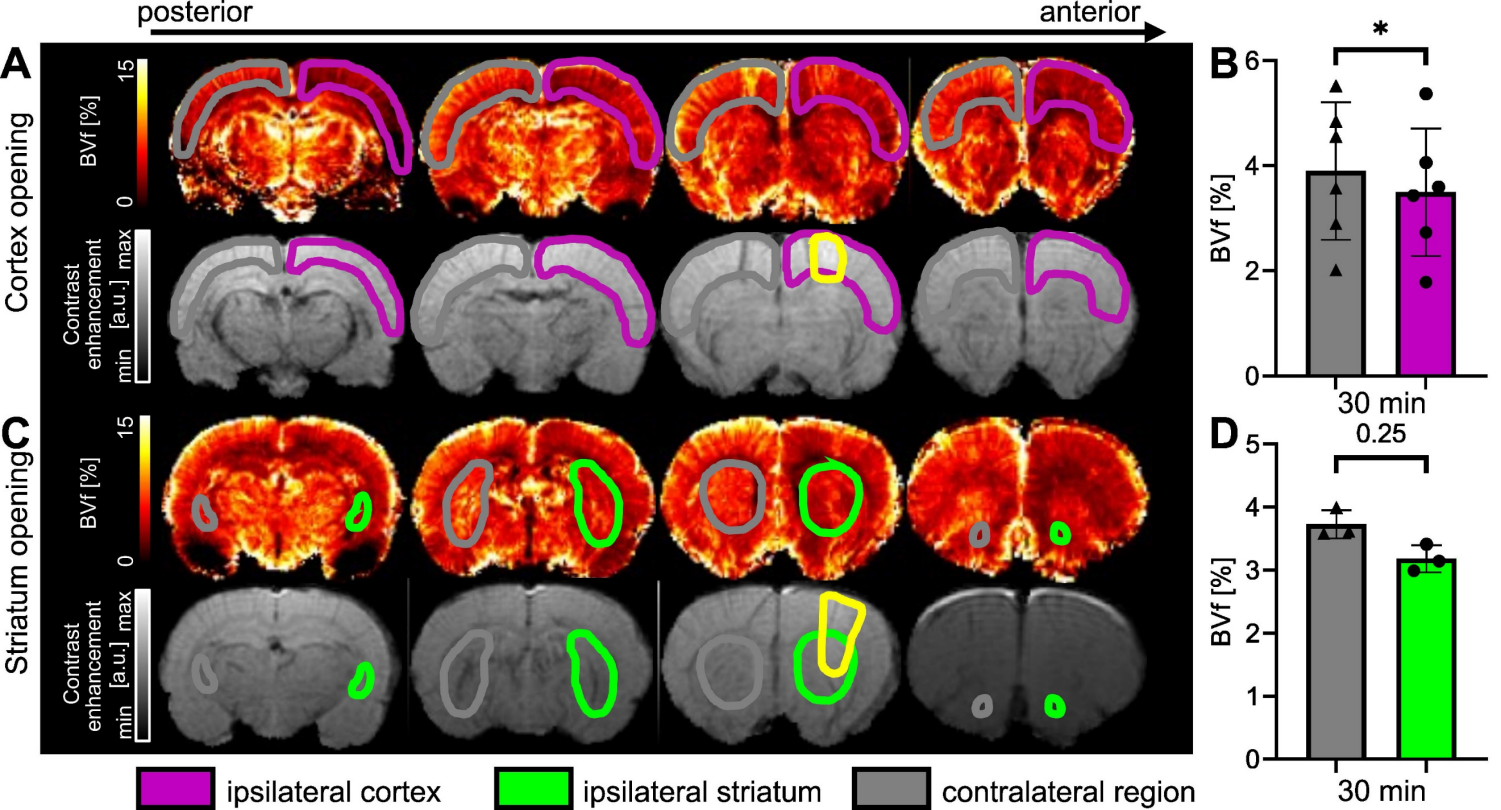
Examples of BVf maps 30 min after BBB opening in the cortex (**A**) and in the striatum (**C**), together with associated T1-w images following Gd-DOTA injection. The BBB-opened area is delineated in yellow. BVf was quantified 30 min after FUS in the cortex (**B**) and in the striatum (**D**). The data were analysed using a two-tailed non-parametric test (Wilcoxon) (*p < 0.05). The results are expressed as mean ± S.D.

The measurements of BVf in the cortex targeted by FUS (ipsilateral) and in the contralateral cortex were calculated from MGE images acquired before and after injection of USPIO nanoparticles 31. The results, expressed as a percentage of blood occupying the voxel, are shown in Figure 8. Figure 8A illustrates the reduction in BVf values in the targeted cortex extending beyond the open area (highlighted in yellow on the T1-w after Gd-DOTA injection). It should be noted that Gd-DOTA was injected after USPIO, and this does not prevent the observation of contrast enhancement of Gd-DOTA on T1-w images 40. A significant reduction in BVf was measured (*p* = 0.0313). The average reduction in BVf was 13% (3.9 ± 1.3% in the contralateral cortex and 3.4 ± 1.2% in the ipsilateral cortex) (Figure 8B). To evaluate the response in the striatum, a further set of three animals was employed (Figure 8C). No significant difference was observed when the BBB was opened in the striatum, with an average BVf of 3.18 ± 0.2% in the ipsilateral striatum and 3.73 ± 0.2 % in the contralateral striatum (Figure 8D). Consequently, the BVf was found to be reduced by 15% in the ipsilateral region, thereby confirming vasoconstriction across the entire area.

### Cellular impact of the procedure: evaluation of neuroinflammation markers

The expression of two neuroinflammation markers, GFAP and Iba1, was analysed to evaluate the brain tissue response to the FUS procedure and to investigate potential correlations with the measured haemodynamic changes. Additionally, vessels were stained with the SMI71 marker and no differences were found 5 days after the FUS treatment. This suggests that 30 min following the measured vasoconstriction, the blood vessels returned to their original shape (Figure S5).

**Figure 9:**
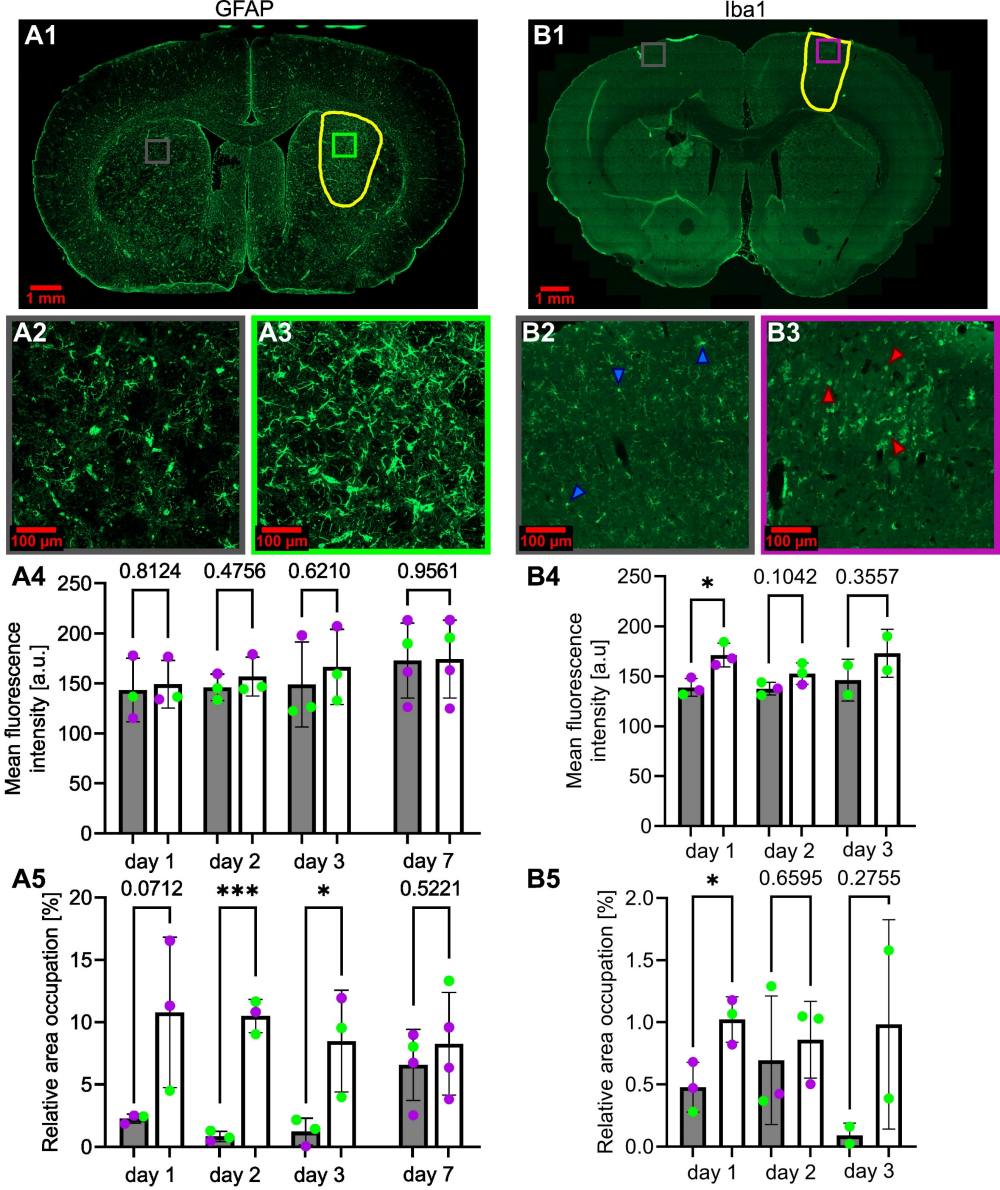
Activation of astrocytes (**A1**) and microglia (**B1**), respectively, 3 days and 1 day after FUS-induced BBB opening. The BBB-opened area is delineated in yellow. Amplified images of the contralateral (grey square) and ipsilateral (purple for cortex, green for striatum) regions (**A2-3**, **B2-3**) are also shown. The blue (**B2**) and red (**B3**) arrows highlight microglia cells in their typical basal state (**B2**) and in an activated state (**B3**). The evolution over days after BBB opening of the mean fluorescence intensity of GFAP (**A4**) and Iba1 (**B4**) and of the relative area occupation of GFAP (**A5**) and Iba1 (**B5**) is also shown. For every bar plot, each point represents the value averaged on at least three slices per subject. The green dots represent animals that received the treatment in the striatum, while the purple dots represent those who received a cortical treatment. A two-tailed unpaired test was performed on the data (*p < 0.05, **p < 0.01, ***p < 0.001). The results are expressed as mean ± S.D.

Figure 9, panel A, presents the evolution of the GFAP marker following BBB opening, while panel B presents the evolution of the Iba1 marker. ROIs were drawn at the location of the BBB opening, and measurements of the Iba1 and GFAP markers were pooled together, regardless of whether the cortex or the striatum were permeabilized. Additionally, the inflammation does not appear to depend on the location of the deposited FUS energy.

Following the FUS treatment, a large astrocytic activation was observed in the ipsilateral region (Figure 9A.3) compared to the control region (Figure 9A.2). This astrocytic activation was observed in the BBB-opened area, but not in the area with reduced CBF (not shown). The mean fluorescence intensity was not significantly different between the contralateral and the ipsilateral regions (Figure 9A.4). However, the relative area occupation was significantly increased in the ipsilateral region two days (p<0.001) and three days (*p*<0.05) after the treatment (Figure 9A.5). Astrocytic activation tends to return to baseline levels one week after the FUS-induced BBB opening.

Figures 9B.2 and B.3 illustrate a change in microglia shape between the ipsilateral and contralateral regions. In Figure 9B.2, the microglia exhibits a ramified shape indicated by the blue arrows. In contrast, the ipsilateral region displays a rounded shape, as highlighted by the red arrows in Figure 9B.3. The mean fluorescence intensity and relative area occupation of segmented pixels were quantified by image processing in the ipsilateral region compared to the contralateral. A significant increase in the ipsilateral region was observed one day after the BBB opening, with a subsequent decrease on the second- and third-days post-treatment (Figure 9B.4). Similarly, an increase in the relative area occupation of microglia was significant on the first day after the BBB opening, with a subsequent decrease in the following days (Figure 9B.5).

## Discussion

Understanding the side effects associated with FUS-mediated BBB opening is crucial for developping effective therapeutic strategies that rely on localised drug delivery to the brain. In our study, we observed that, despite the absence of tissue damage, both blood flow and the blood volume were significantly reduced for at least one hour in an extended region surrounding the BBB opening area. While perfusion normalised within 24 h post-FUS, transient inflammation persisted for several days in the targeted tissue. Additionally, we introduced a method for monitoring the MBs cavitation status during FUS treatment, enhancing the robustness of our experiments.

The BBB opening was performed with real-time cavitation monitoring and homemade lipidic MBs. We chose an experimental setup with MBs and a mechanical index similar to those used in previous studies 5,41, and all procedures resulted in BBB opening. The mechanical index (PNP divided by the square root of the acoustic frequency) was 0.5 in this study. According to Chu *et al*. 5, this confirmed the safety of the FUS procedure. Indeed, with this setup, 95% of the BBB opening procedures resulted in neither oedema nor bleeding, as assessed by MRI. It is noteworthy that the quantification of inertial cavitation events using IUD enabled the differentiation between animals that underwent excessive FUS treatment and developed lesions and those that did not. While this was not the primary objective of the study, these findings are encouraging and warrant further investigation, particularly with a larger number of animals to demonstrate the full potential of this approach. A specific study of critical cavitation situations could confirm and improve the control of this behaviour as well as our understanding. Additionally, future developments should include closed-loop cavitation control to prevent lesions. Consequently, the safety of the BBB opening procedure could be enhanced, thereby improving animal outcomes 3.

Brain perfusion was measured between 30 min and 24 h after FUS treatment. While the CBF in the contralateral hemisphere was normal and comparable to previous literature reports 34-39, the CBF in the hemisphere exposed to FUS experienced a 38% reduction within 30 min after FUS treatment. This reduction was lower than that observed in a stroke model, where a reduction of approximately 70% has been reported 42. The magnitude of the CBF reduction observed after FUS treatment is consistent with the observation that no change in ADC was observed, which typically occurs in stroke models with larger CBF reduction 42. Such a reduction has previously been reported by Todd *et al*. 15 between one and two hours after FUS treatment. Over time, CBF in our study recovered. At 90 min after FUS treatment, the difference was no longer significant, possibly due to a small number of animals. At 24 h, no difference could be observed.

To further understand the origin of this CBF reduction, we also measured BVf 30 min after FUS. BVf was reduced by only 15% compared to 50% in the stroke model. A reduction in vessel diameter after FUS, in line with a reduction in BVf, has previously been observed using intravital microscopy 43. A larger reduction in blood flow than in blood volume may be explained by a reduction in the flow entering the observed area. Interestingly, the reduction in CBF and BVf maps were spatially homogeneous, suggesting that all vessels in the area underwent vasoconstriction, which might have been more pronounced at the level of larger feeding arteries. Moreover, the area in which perfusion parameters were reduced extended beyond the FUS target, as evidenced by the extravasation of the contrast agent. This occurred when the target was in the cortex (the entire cortex exhibited reduced perfusion) and when it was in the striatum (the entire striatum became hypoperfused). This spatial mismatch between the extension of the US target and that of the perfusion reduction has been previously reported in the literature, without the use of a focused transducer or cavitation monitoring 17.

The origin of this discrepancy could be attributed to retroactive vasoconstriction, based on the retroactive vasodilation mechanism described by Schaeffer and Iadecola 44. An alternative mechanism could be the slow propagating cortical spreading depressions described by Leaõ 45, which have been reported after traumatic brain injury (TBI) 46 and stroke 47,48. Altogether, it can be stated that, even in the absence of MB cavitation and the use of a focused transducer, a transient reduction in perfusion occurs and extends beyond the FUS target. This reduction does not lead to tissue oedema, as monitored using ADC. Further experiments, such as transcranial ultrasound localisation microscopy 49 or cortical electrical activity recording 50 could be performed to further characterise the reduction in brain perfusion that occurs after FUS.

At the cellular level, some changes occurred during the first three days after FUS. We quantified the peak of astrocytic activation at day three post-BBB opening, a result very similar to that reported by Jordão *et al*. 51. Moreover, a significant increase in microglia recruitment was observed, suggesting that the neuroprotective role of the CNS was triggered. The activation peak was observed at day one after FUS. In contrast, several studies reviewed by Todd *et al*. 52 reported activations at 1, 6 and 24 h post-BBB opening. Both astrocytes and microglia returned to normal levels one week after FUS. It is noteworthy that this neuroinflammation was observed only in the FUS target and was not observed in the extended area where perfusion was reduced. This suggests that inflammation is triggered by the BBB opening. However, it cannot be excluded that the perfusion reduction also contributes to this effect.

When extrapolating our findings to humans, a key difference lies in the fact that our study was conducted under anaesthesia, which reduces the cerebral metabolic rate of oxygen 53. Moreover, isoflurane anaesthesia tends to elevate CBF. Considering that a decrease in oxygen demand and an increase in oxygen supply may confer some degree of protection against reduced perfusion, our study might underestimate the consequences of the FUS-induced perfusion reduction. Conversely, it has been reported that FUS-mediated BBB opening transiently impedes axonal conduction, thereby lowering neuronal activity and potentially protecting the tissue against a perfusion decrease 54. Further investigations could involve awake animal studies or employ anaesthesia protocols that yield CBF values akin to those observed in the awakened state. Given the reduction in blood flow and the absence of blood vessel alteration (Figure S5), it would also be of interest to explore whether angiogenic processes are triggered, as suggested by Kovacs *et al*. 18. This could also imply an assessment of the expression of HIF-1a or VEGF, shortly after FUS.

In summary, FUS-mediated BBB opening induces diffuse vasoconstriction beyond the targeted area, resulting in a transient reduction in both CBF and BVf. However, this reduction is insufficient to cause tissue lesions in our experimental setup. While transient markers of tissue inflammation may be observed at the FUS-targeted area, they are not observed outside this area. These findings emphasise the need of caution regarding tissue perfusion when considering the translation of FUS-mediated BBB opening to human applications.

## Abbreviations

ADC: apparent diffusion coefficient
BBB: blood-brain barrier
BVf: blood volume fraction
CA: contrast agent
CBF: cerebral blood flow
CNS: central nervous system
DTI: diffusion tensor imaging
EPI: echo planar imaging
FOV: field of view
FUS: focused ultrasound
FWHM: full width at half maximum
Gd-DOTA: Dotarem
GFAP: glial fibrillary acidic protein
H&E: hematoxylin & eosin
HEPES: 4-(2-hydroxyethyl)-1-piperazineethanesulfonic acid
IL-1: interleukin 1
IL-18: interleukin 18
IV: intravenous
MBs: microbubbles
MGE: multi-gradient echo
MRgFUS: magnetic resonance guided focused ultrasound
MRI: magnetic resonance imaging
NO: nitric oxide
NVU: neurovascular unit
pCASL: pseudo-continuous arterial spin labelling
PNP: peak negative pressure
RARE: rapid acquisition with relaxation enhancement
RBCs: red blood cells
ROI: region of interest
S1HL: somatosensory cortex of the hindlimb
S.D.: standard deviation, T1-w: T1-weighted
T2-w: T2-weighted
TE: echo time
Tis: inversion times
TNF-α: tumor necrosis factor alpha
TR: relaxation time
USPIO: ultra small paramagnetic iron oxide
