# Supplementary material for: FUS-mediated BBB opening leads to transient perfusion decrease and inflammation without acute or chronic brain lesion
Source: Theranostics. 2024 Jul 2;14(10):4147–60. doi: 10.7150/thno.96721 (PMC11234282; doi:10.7150/thno.96721)
Supplement: Supplementary file 1 — Supplementary figures. [file thnov14p4147s1.pdf]

FUS-mediated BBB opening leads to transient perfusion decrease and inflammation without acute or chronic brain lesion

Sébastien Rigollet[1, 2], Claire Rome[2], Thomas Ador[3, 4], Erik Dumont[1], Chantal Pichon[3, 4, 5], Anthony Delalande[3, 4], Emmanuel L. Barbier[2, 6#], Vasile Stupar[2, 6]

doi:10.7150/thno.96721

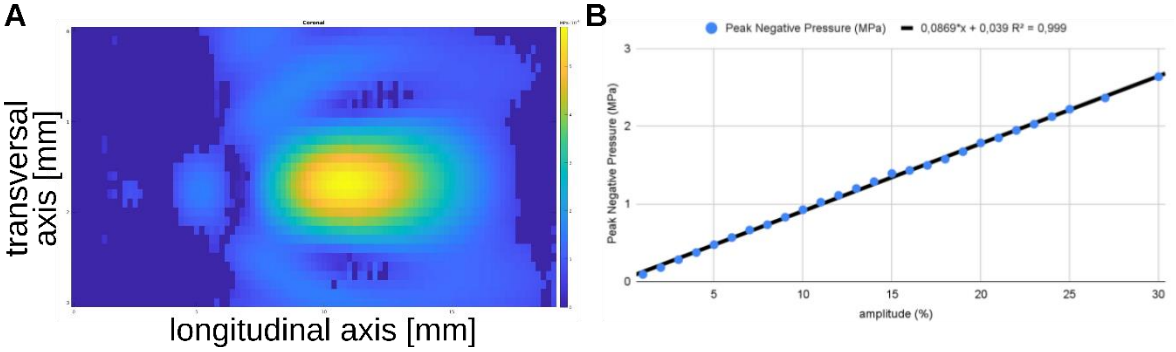

Figure S1. (**A**) measured focal point in a degassed water tank with a needle hydrophone and (**B**) calibration curve of the peak negative pressure depending on the amplitude of the FUS pulse.

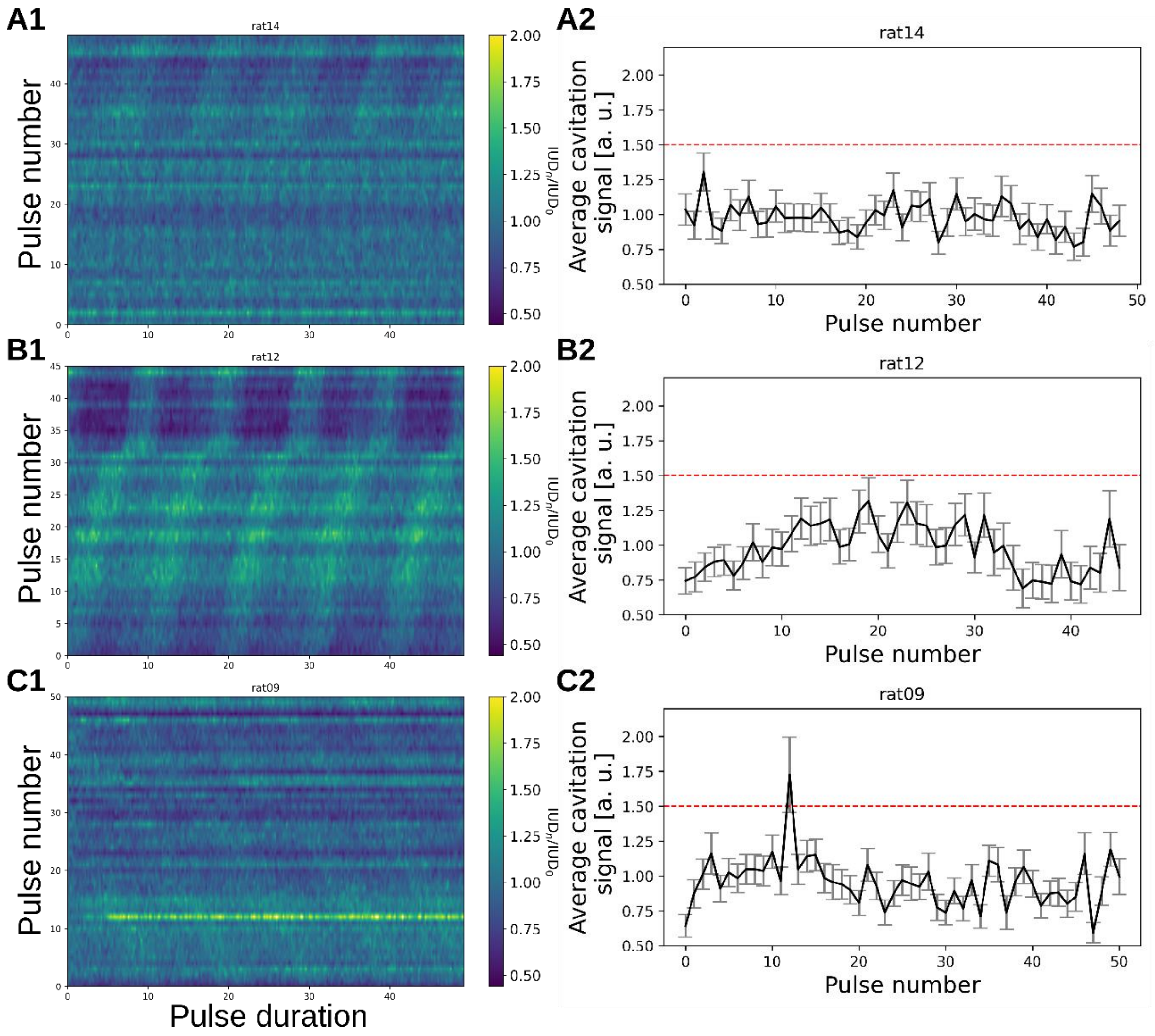

Figure S2. Example of the evolution of the relative IUD over every pulse of the treatment for soft (**A1**), mild (**B1**) and hard (**C1**) classification treatment. The corresponding average and standard deviation cavitation signal for the three classification cases with the red dashed line representing the inertial threshold (**A2**, **B2**, **C2**).

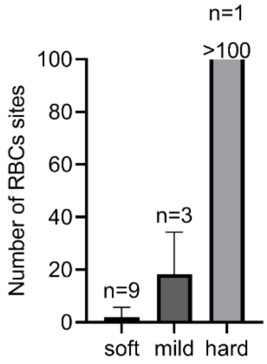

Figure S3. number of RBCs sites analysed on H&E images for each cavitation-based classification group. The results are expressed as mean ± S.D.

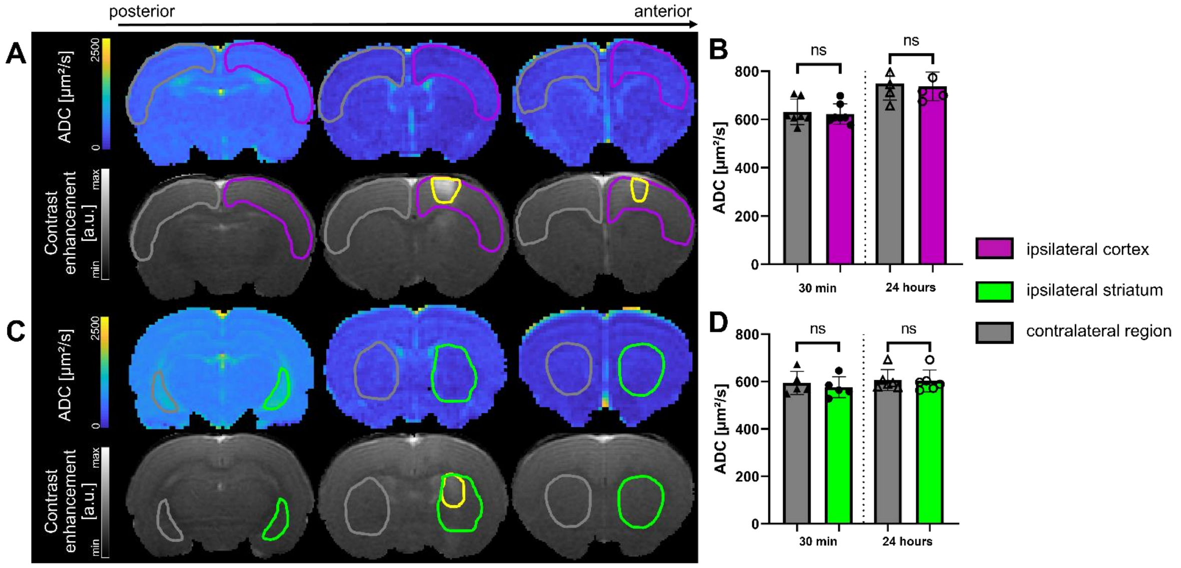

Figure S4. Examples of ADC maps 30 minutes after BBB opening in the cortex (**A**) and striatum (**C**), and associated T1-w images after Gd-DOTA injection. The opened area is delineated in yellow. ADC 30 minutes and 24 hours after FUS in the cortex (**B**) and in the striatum (**D**). Data were analysed using a two-tailed non-parametric test (Wilcoxon) (*p < 0.05; **p < 0.01; ***p < 0,001; ns, non-significant). Results are expressed as mean ± S.D.

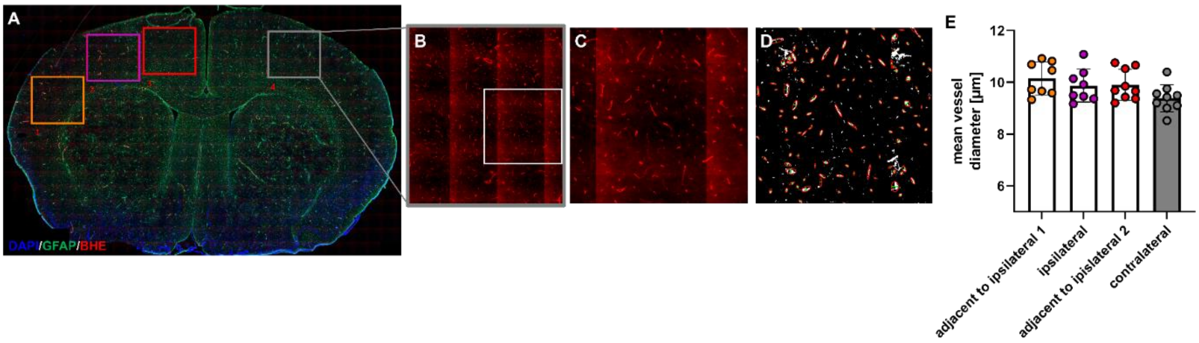

Figure S5. (**A**) representative micrograph of DAPI, GFAP and BBB markers with 2 adjacent ROI (orange and red). (**B**) and (**C**) zoom at different levels on the contralateral cortex. (**D**) example from image segmentation and diameter analysis script. (**E**) mean vessel diameter of 2 adjacent ipsilateral ROI at the left and the right of the ipsilateral region. Results are expressed as mean± S.D.
